# Crystal structure of *N*-[(2*S*,5*R*)-4-oxo-2,3-diphenyl-1,3-thia­zinan-5-yl]acetamide 0.375-hydrate

**DOI:** 10.1107/S2056989014026425

**Published:** 2015-01-01

**Authors:** Hemant P. Yennawar, Harnoor Singh, Lee J. Silverberg

**Affiliations:** a Department of Chemistry, Pennsylvania State University, University Park, PA 16802, USA; b Pennsylvania State University, Schuylkill Campus, 200 University Drive, Schuylkill Haven, PA 17972, USA

**Keywords:** crystal structure, thia­zine ring, boat, half-chair, 1,3-thia­zin-4-one, hydrogen bonding

## Abstract

The asymmetric unit of the title compound, C_18_H_18_N_2_O_2_S.0.375H_2_O, has two independent organic mol­ecules (*A* and *B*) and 3/4 of a water mol­ecule distributed over three sites. In mol­ecule *A*, the 1,3-thia­zine ring is in a boat conformation, with the C atoms at the 2- and 5-positions out of the plane. The angle between the two phenyl rings is 51.70 (12)°. In mol­ecule *B*, the thia­zine ring is in a half-chair conformation, with the S atom forming the back of the half-chair. The angle between the two phenyl rings is 84.44 (14)°. The *A* mol­ecule features an intra­molecular N—H⋯O hydrogen bond, which closes an *S*(5) ring motif. In the crystal, the corresponding N—H grouping of the *B* mol­ecule participates in an inter­molecular hydrogen bond to the *A* mol­ecule. The *A* mol­ecule participates in a C—H⋯O inter­action back to the *B* mol­ecule, whilst the *B* mol­ecule features an intra­molecular C—H⋯O link, which generates an *S*(10) loop.

## Abstract 2

The crystal structure of the title compound displays boat and half-chair configurations of the thia­zine ring.

## Chemical context   

In a recent paper, we reported the 2,4,6-tripropyl-1,3,5,2,4,6-trioxatri­phospho­rinane-2,4,6-trioxide (T3P)-promoted cyclization of *N*-[phenyl­methyl­idene]aniline with 3-sulfanyl­propanoic acid to produce 2,3-diphenyl-2,3,5,6-tetra­hydro-4*H*-1,3-thia­zin-4-one (Yennawar & Silverberg, 2014 ▸). As noted before (Yennawar *et al.*, 2014 ▸), prior to this, the *N*-aryl compounds had not easily been prepared by condensation of imines with thio­acids. With respect to the thio­acid, the use of a homochiral cysteine derivative is desirable because, along with putting a functional group on the ring, it creates a second chiral center at the 5-position of the thia­zinone, potentially allowing the separation of two diastereomers into *cis* and *trans* homochiral heterocycles. A condensation of *N*-acetyl­cysteine with two very active (C*X*_3_)_2_C=NH imines has been reported (Raasch, 1974 ▸), giving a thia­zinone with one chiral center. Although a search of 2,3,5,6-tetra­hydro-4*H*-1,3-thia­zin-4-ones with a nitro­gen atom at the 5-position and carbon atoms at positions 2 and 3 found 156 compounds, there were only two compounds with an aryl group at the 3-position and both involved a more complex bridged structure synthesized by a cyclo­addition route (Potts, *et al.*, 1974 ▸). Herein we report the T3P-promoted cyclization of *N*-[phenyl­methyl­idene]aniline with *N*-acetyl-l-cysteine. One major product arose along with at least three minor products, as determined by NMR spectroscopy. The major product was isolated by column chromatography followed by recrystallization. The structure is reported as the title compound here. The minor products have not yet been satisfactorily isolated. As reported here, the major product is the *cis* diastereomer.
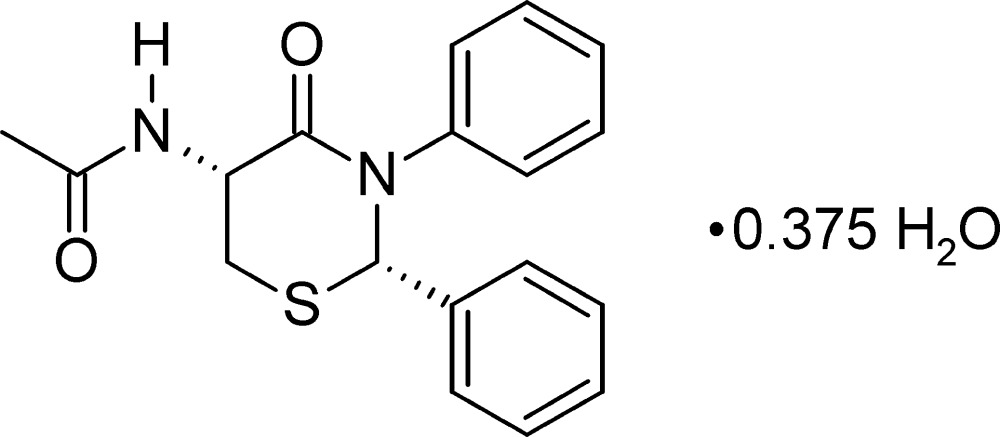


## Structural commentary   

**Figure 1:**
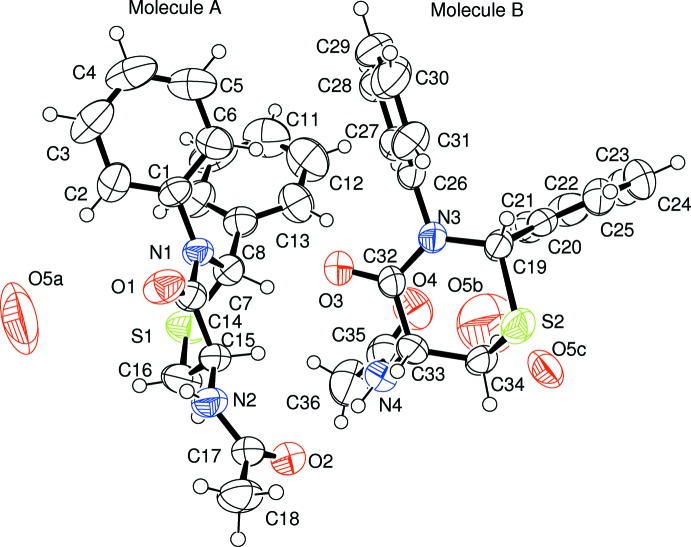
*ORTEP* view of the title compound. Displacement ellipsoids are drawn at the 50% probability level.

The two independent organic mol­ecules in the asymmetric unit exhibit different geometries for the thia­zine ring (Fig. 1 ▸). In mol­ecule *A*, the ring takes a boat configuration with the groups at the 2- and 5-positions *pseudo*-equatorial and the hydrogens at these positions within 1.993 Å of each other. The stability gained by having both groups *pseudo*-equatorial must offset the higher energy expected in a boat conformation. The dihedral angle between the C1- and C8-benzene rings is 51.7 (2)°. An intra­molecular N2—H2*N*⋯O1 hydrogen bond is observed, which closes an *S*(5) ring.

In mol­ecule *B*, the thia­zine ring adopts a half-chair conformation. The groups at the 2- and 5-positions cannot readily be defined as *pseudo*-axial or *pseudo*-equatorial, but the phenyl ring at the 2-position is closer to axial, while the amide group at the 5-position is closer to equatorial. The dihedral angle between the phenyl rings (C20–C25 and C26–C31) is 84.4 (2)°. This conformation is similar to that observed for 2,3-diphenyl-2,3,5,6-tetra­hydro-4*H*-1,3-thia­zin-4-one (Yennawar & Silverberg, 2014 ▸). Mol­ecule *B* features an intra­molecular C21—H21⋯O4 link, which generates an *S*(10) loop.

The residual electron density suggested several solvent mol­ecule sites but only with partial occupancies. The best model fixed the occupancy for each of the three water-mol­ecule sites at 0.25.

## Supra­molecular features   

**Figure 2:**
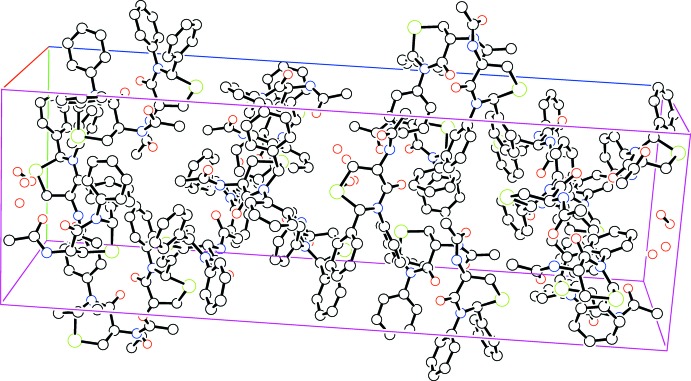
The crystal packing of the title compound.

In the crystal, the N—H grouping of mol­ecule *B* (corres­ponding to the one involved in the intra­molecular N2—H2*N*⋯O1 hydrogen bond in mol­ecule *A*) participates in an inter­molecular N4—H4*N*⋯O2 hydrogen bond to mol­ecule *A* (Table 1 ▸). Mol­ecule *A* participates in a C7—H7⋯O3 inter­action back to mol­ecule *B*. The crystal packing is shown in Fig. 2 ▸.

## Synthesis and crystallization   

A two-necked 25 ml round-bottom flask was oven-dried, cooled under N_2_, and charged with a stir bar and *N*-benzyl­ideneaniline (1.087 g, 6 mmol). Tetra­hydro­furan (2.3 ml) was added, the solid dissolved, and the solution was stirred. Pyridine (1.95 ml, 24 mmol) was added and then *N*-acetyl-l-cysteine (6 mmol, 0.9824 g) was added. Finally, 2,4,6-tripropyl-1,3,5,2,4,6-trioxatri­phospho­rinane-2,4,6-trioxide (T3P) in 2-methyl­tetra­hydro­furan (50 weight%; 7.1 ml, 12 mmol) was added. The reaction was stirred at room temperature. TLC (EtOAc) after one day showed the reaction was complete, with two product spots, but the reaction was allowed to stir another 13 days. The mixture was poured into a separatory funnel with di­chloro­methane and distilled water. The layers were separated and the aqueous was then extracted twice with di­chloro­methane. The organics were combined and washed with saturated sodium bicarbonate and then saturated sodium chloride. The organic was dried over sodium sulfate, and concentrated under vacuum to a solid. The crude was chromatographed on 30 g flash silica gel, eluting with 50% ethyl acetate/hexa­nes and 100% ethyl acetate. Fractions containing the larger, more polar spot on TLC were combined, concentrated under vacuum, recrystallized from ethyl acetate/hexa­nes, and rinsed with ethanol to give light-yellow crystals of *N*-[(2*S*, 5*R*)-4-oxo-2,3-diphenyl-1,3-thia­zinan-5-yl]acetamide (0.2702 g, 13.8%). m.p.: 460–463 K. *R*_f_ = 0.24 (EtOAc). Colourless cuboids were grown by slow evaporation from 2-propanol. The fractions containing the other TLC spot [*R*_f_ = 0.33 (EtOAc)] showed four different compounds by NMR, including the title compound.

## Refinement   

Crystal data, data collection and structure refinement details are summarized in Table 2 ▸. The hydrogen atoms bound to the nitro­gen atom was located in the difference Fourier map and refined isotropically. The C-bound H atoms were geometrically placed with C—H = 0.93–0.97 Å, and refined as riding with *U*_iso_(H) = 1.2U_eq_(C). The three solvent mol­ecule sites were given occupancy of 0.25 each, as that proved to be the best way to account for the residual electron density.

## Supplementary Material

Crystal structure: contains datablock(s) I. DOI: 10.1107/S2056989014026425/hb7315sup1.cif

Structure factors: contains datablock(s) I. DOI: 10.1107/S2056989014026425/hb7315Isup2.hkl

Supporting information file. DOI: 10.1107/S2056989014026425/hb7315Isup3.mol

Supporting information file. DOI: 10.1107/S2056989014026425/hb7315Isup4.cml

CCDC reference: 1037009

Additional supporting information:  crystallographic information; 3D view; checkCIF report

## Figures and Tables

**Table 1:** Hydrogen-bond geometry (, )

*D*H*A*	*D*H	H*A*	*D* *A*	*D*H*A*
N2H2*N*O1	0.88(6)	2.22(5)	2.678(4)	113(5)
N4H4*N*O2	0.90(4)	2.03(4)	2.899(5)	162(4)
C7H7O3	0.98	2.50	3.241(4)	132
C21H21O4	0.93	2.48	3.375(5)	162

**Table 2:** Experimental details

Crystal data	
Chemical formula	2C_18_H_18_N_2_O_2_S0.75OH_2_O
*M*_r_	664.81
Crystal system, space group	Tetragonal, *P*4_1_2_1_2
Temperature (K)	298
*a*, *c* ()	13.3438(12), 40.237(7)
*V* (^3^)	7164.6(16)
*Z*	8
Radiation type	Mo *K*
(mm^1^)	0.19
Crystal size (mm)	0.28 0.25 0.20

Data collection	
Diffractometer	Bruker SMART APEX CCD
Absorption correction	Multi-scan (*SADABS*; Bruker, 2001 ▸)
*T*_min_, *T*_max_	0.948, 0.962
No. of measured, independent and observed [*I* > 2(*I*)] reflections	56028, 8891, 6254
*R*_int_	0.049
(sin /)_max_ (^1^)	0.667

Refinement	
*R*[*F* ^2^ > 2(*F* ^2^)], *wR*(*F* ^2^), *S*	0.066, 0.216, 1.05
No. of reflections	8891
No. of parameters	448
H-atom treatment	H atoms treated by a mixture of independent and constrained refinement
_max_, _min_ (e ^3^)	0.75, 0.48
Absolute structure	Flack (1983 ▸), 3769 Friedel pairs
Absolute structure parameter	0.1(1)

Computer programs: *SMART* and *SAINT* (Bruker, 2001 ▸), *SHELXS97* and *SHELXL97* (Sheldrick, 2008 ▸), *XSHELL* (Bruker, 2001 ▸) and *ORTEP-3 for Windows* (Farrugia, 2012 ▸).

## supplementary crystallographic information

### Crystal data

**Table:**

2C_18_H_18_N_2_O_2_S·0.75OH_2_O	*D*_x_ = 1.233 Mg m^−^^3^
*M**_r_* = 664.81	Melting point: 451(2) K
Tetragonal, *P*4_1_2_1_2	Mo *K*α radiation, λ = 0.71073 Å
Hall symbol: P 4abw 2nw	Cell parameters from 6252 reflections
*a* = 13.3438 (12) Å	θ = 2.2–26.4°
*c* = 40.237 (7) Å	µ = 0.19 mm^−^^1^
*V* = 7164.6 (16) Å^3^	*T* = 298 K
*Z* = 8	Cube, colorless
*F*(000) = 2800	0.28 × 0.25 × 0.20 mm

### Data collection

**Table:**

Bruker SMART APEX CCD diffractometer	8891 independent reflections
Radiation source: fine-focus sealed tube	6254 reflections with *I* > 2σ(*I*)
Graphite monochromator	*R*_int_ = 0.049
Detector resolution: 8.34 pixels mm^-1^	θ_max_ = 28.3°, θ_min_ = 1.6°
φ and ω scans	*h* = −16→17
Absorption correction: multi-scan (*SADABS*; Bruker, 2001)	*k* = −17→16
*T*_min_ = 0.948, *T*_max_ = 0.962	*l* = −53→53
56028 measured reflections	

### Refinement

**Table:**

Refinement on *F*^2^	Secondary atom site location: difference Fourier map
Least-squares matrix: full	Hydrogen site location: inferred from neighbouring sites
*R*[*F*^2^ > 2σ(*F*^2^)] = 0.066	H atoms treated by a mixture of independent and constrained refinement
*wR*(*F*^2^) = 0.216	*w* = 1/[σ^2^(*F*_o_^2^) + (0.140*P*)^2^] where *P* = (*F*_o_^2^ + 2*F*_c_^2^)/3
*S* = 1.05	(Δ/σ)_max_ = 0.008
8891 reflections	Δρ_max_ = 0.75 e Å^−^^3^
448 parameters	Δρ_min_ = −0.48 e Å^−^^3^
0 restraints	Absolute structure: Flack (1983), 3769 Friedel pairs
Primary atom site location: structure-invariant direct methods	Absolute structure parameter: 0.10 (10)

### Special details

**Table:**

Experimental. Absorption correction: SADABS was used for absorption correction. R(int) was 0.0331 before and 0.0128 after correction. The Ratio of minimum to maximum transmission is 0.8482. The λ/2 correction factor is 0.0015.The data collection nominally covered a full sphere of reciprocal space by a combination of 4 sets of ω scans each set at different φ and/or 2θ angles and each scan (10 s exposure) covering -0.300° degrees in ω. The crystal to detector distance was 5.82 cm.
Geometry. All e.s.d.'s (except the e.s.d. in the dihedral angle between two l.s. planes) are estimated using the full covariance matrix. The cell e.s.d.'s are taken into account individually in the estimation of e.s.d.'s in distances, angles and torsion angles; correlations between e.s.d.'s in cell parameters are only used when they are defined by crystal symmetry. An approximate (isotropic) treatment of cell e.s.d.'s is used for estimating e.s.d.'s involving l.s. planes.
Refinement. Refinement of *F*^2^ against ALL reflections. The weighted *R*-factor *wR* and goodness of fit *S* are based on *F*^2^, conventional *R*-factors *R* are based on *F*, with *F* set to zero for negative *F*^2^. The threshold expression of *F*^2^ > σ(*F*^2^) is used only for calculating *R*-factors(gt) *etc*. and is not relevant to the choice of reflections for refinement. *R*-factors based on *F*^2^ are statistically about twice as large as those based on *F*, and *R*- factors based on ALL data will be even larger.

### Fractional atomic coordinates and isotropic or equivalent isotropic displacement parameters (Å^2^)

**Table:**

	*x*	*y*	*z*	*U*_iso_*/*U*_eq_	Occ. (<1)
O5A	0.0057 (15)	0.053 (2)	0.2410 (7)	0.178 (10)	0.25
C1	0.2504 (3)	−0.0827 (2)	0.18198 (8)	0.0489 (7)	
C2	0.1531 (3)	−0.1098 (3)	0.18700 (10)	0.0651 (9)	
H2	0.1094	−0.0680	0.1985	0.078*	
C3	0.1199 (4)	−0.2031 (4)	0.17437 (13)	0.0878 (15)	
H3	0.0530	−0.2212	0.1768	0.105*	
C4	0.1842 (5)	−0.2668 (3)	0.15865 (14)	0.0923 (16)	
H4	0.1618	−0.3286	0.1510	0.111*	
C5	0.2808 (4)	−0.2392 (3)	0.15436 (11)	0.0800 (13)	
H5	0.3247	−0.2825	0.1436	0.096*	
C6	0.3156 (3)	−0.1476 (3)	0.16574 (9)	0.0625 (9)	
H6	0.3822	−0.1297	0.1625	0.075*	
C7	0.3648 (3)	0.0213 (3)	0.21946 (8)	0.0509 (7)	
H7	0.4174	0.0648	0.2105	0.061*	
C8	0.4137 (3)	−0.0748 (3)	0.22965 (8)	0.0562 (8)	
C9	0.3653 (3)	−0.1439 (3)	0.24980 (10)	0.0706 (10)	
H9	0.3010	−0.1323	0.2579	0.085*	
C10	0.4193 (5)	−0.2344 (3)	0.25755 (12)	0.0907 (16)	
H10	0.3891	−0.2829	0.2708	0.109*	
C11	0.5109 (5)	−0.2497 (4)	0.24620 (14)	0.0932 (16)	
H11	0.5439	−0.3088	0.2517	0.112*	
C12	0.5571 (4)	−0.1833 (5)	0.22722 (15)	0.0994 (17)	
H12	0.6211	−0.1966	0.2192	0.119*	
C13	0.5102 (3)	−0.0938 (4)	0.21928 (11)	0.0739 (10)	
H13	0.5442	−0.0461	0.2068	0.089*	
C14	0.2462 (2)	0.0994 (2)	0.18183 (8)	0.0499 (7)	
C15	0.2878 (3)	0.1931 (2)	0.19744 (9)	0.0545 (7)	
H15	0.3607	0.1932	0.1945	0.065*	
C16	0.2647 (4)	0.1940 (3)	0.23457 (10)	0.0724 (10)	
H16A	0.1927	0.1975	0.2377	0.087*	
H16B	0.2940	0.2535	0.2444	0.087*	
C17	0.2967 (3)	0.3685 (2)	0.17969 (9)	0.0577 (8)	
C18	0.2486 (4)	0.4517 (3)	0.16103 (11)	0.0772 (12)	
H18A	0.2521	0.5121	0.1739	0.116*	
H18B	0.1798	0.4353	0.1568	0.116*	
H18C	0.2830	0.4614	0.1403	0.116*	
C19	0.6796 (2)	0.1320 (2)	0.09363 (8)	0.0481 (7)	
H19	0.6662	0.1120	0.0706	0.058*	
C20	0.7810 (2)	0.0882 (2)	0.10234 (9)	0.0516 (7)	
C21	0.8070 (3)	0.0618 (3)	0.13434 (10)	0.0612 (8)	
H21	0.7602	0.0671	0.1514	0.073*	
C22	0.9023 (3)	0.0278 (3)	0.14103 (15)	0.0826 (13)	
H22	0.9196	0.0100	0.1626	0.099*	
C23	0.9725 (3)	0.0197 (4)	0.11586 (16)	0.0866 (15)	
H23	1.0374	−0.0012	0.1207	0.104*	
C24	0.9467 (3)	0.0419 (4)	0.08473 (16)	0.0900 (15)	
H24	0.9933	0.0340	0.0677	0.108*	
C25	0.8515 (3)	0.0765 (3)	0.07738 (12)	0.0693 (10)	
H25	0.8347	0.0921	0.0555	0.083*	
C26	0.5686 (2)	−0.0093 (2)	0.10451 (8)	0.0492 (7)	
C27	0.5836 (3)	−0.0874 (3)	0.12672 (11)	0.0614 (8)	
H27	0.6106	−0.0748	0.1476	0.074*	
C28	0.5582 (3)	−0.1838 (3)	0.11770 (13)	0.0726 (11)	
H28	0.5669	−0.2361	0.1327	0.087*	
C29	0.5199 (4)	−0.2029 (3)	0.08654 (14)	0.0817 (13)	
H29	0.5027	−0.2681	0.0806	0.098*	
C30	0.5071 (4)	−0.1261 (4)	0.06417 (13)	0.0899 (15)	
H30	0.4823	−0.1396	0.0430	0.108*	
C31	0.5311 (3)	−0.0281 (3)	0.07304 (10)	0.0678 (10)	
H31	0.5220	0.0240	0.0580	0.081*	
C32	0.5461 (2)	0.1382 (2)	0.13924 (8)	0.0490 (7)	
C33	0.5743 (3)	0.2454 (3)	0.14941 (9)	0.0542 (8)	
H33	0.5249	0.2891	0.1387	0.065*	
C34	0.6759 (3)	0.2826 (2)	0.13793 (10)	0.0570 (8)	
H34A	0.7286	0.2442	0.1486	0.068*	
H34B	0.6842	0.3525	0.1439	0.068*	
C35	0.6035 (3)	0.1985 (4)	0.20735 (10)	0.0707 (10)	
C36	0.5771 (5)	0.2170 (5)	0.24334 (11)	0.1019 (17)	
H36A	0.5455	0.2813	0.2455	0.153*	
H36B	0.6371	0.2157	0.2565	0.153*	
H36C	0.5321	0.1657	0.2509	0.153*	
N1	0.28612 (19)	0.01260 (19)	0.19383 (7)	0.0474 (6)	
N2	0.2471 (3)	0.2807 (2)	0.18125 (9)	0.0601 (7)	
H2N	0.193 (5)	0.265 (4)	0.1701 (12)	0.100 (17)*	
N3	0.59653 (19)	0.09217 (19)	0.11341 (7)	0.0481 (6)	
N4	0.5591 (3)	0.2575 (3)	0.18477 (9)	0.0633 (8)	
H4N	0.511 (3)	0.305 (3)	0.1853 (9)	0.053 (10)*	
O1	0.1807 (2)	0.10192 (19)	0.16054 (7)	0.0673 (7)	
O2	0.3791 (2)	0.3763 (2)	0.19349 (8)	0.0734 (7)	
O3	0.47621 (19)	0.09644 (19)	0.15238 (7)	0.0629 (6)	
O4	0.6614 (3)	0.1312 (3)	0.19895 (8)	0.0841 (9)	
S1	0.31262 (10)	0.08417 (8)	0.25589 (2)	0.0726 (3)	
S2	0.68356 (7)	0.26844 (7)	0.09362 (3)	0.0624 (2)	
O5C	0.9973 (9)	0.2040 (12)	0.2631 (3)	0.092 (4)	0.25
O5B	0.8745 (12)	0.1255 (12)	0.2500	0.270 (15)	0.50

### Atomic displacement parameters (Å^2^)

**Table:**

	*U*^11^	*U*^22^	*U*^33^	*U*^12^	*U*^13^	*U*^23^
O5A	0.092 (12)	0.22 (3)	0.22 (3)	0.038 (16)	0.064 (14)	0.00 (2)
C1	0.0553 (18)	0.0383 (15)	0.0533 (15)	−0.0032 (13)	−0.0101 (14)	0.0055 (12)
C2	0.0536 (19)	0.064 (2)	0.078 (2)	−0.0075 (16)	−0.0073 (17)	0.0157 (18)
C3	0.079 (3)	0.070 (3)	0.114 (3)	−0.028 (2)	−0.036 (3)	0.023 (3)
C4	0.115 (4)	0.046 (2)	0.116 (4)	−0.008 (3)	−0.047 (3)	0.002 (2)
C5	0.103 (4)	0.054 (2)	0.083 (3)	0.014 (2)	−0.024 (2)	−0.013 (2)
C6	0.066 (2)	0.061 (2)	0.0607 (19)	0.0083 (17)	−0.0060 (17)	−0.0083 (16)
C7	0.0494 (17)	0.0505 (17)	0.0527 (16)	−0.0001 (13)	−0.0053 (13)	0.0027 (13)
C8	0.059 (2)	0.0545 (19)	0.0552 (16)	−0.0001 (15)	−0.0102 (15)	−0.0019 (14)
C9	0.074 (3)	0.069 (2)	0.069 (2)	−0.0090 (19)	−0.006 (2)	0.0174 (19)
C10	0.132 (5)	0.058 (2)	0.083 (3)	−0.016 (3)	−0.038 (3)	0.017 (2)
C11	0.099 (4)	0.083 (3)	0.097 (3)	0.023 (3)	−0.037 (3)	0.007 (3)
C12	0.081 (3)	0.106 (4)	0.111 (4)	0.033 (3)	−0.023 (3)	0.006 (3)
C13	0.059 (2)	0.089 (3)	0.074 (2)	0.016 (2)	−0.0084 (19)	0.007 (2)
C14	0.0495 (17)	0.0416 (16)	0.0585 (17)	0.0026 (13)	−0.0020 (14)	0.0028 (13)
C15	0.0507 (17)	0.0406 (15)	0.0722 (19)	0.0010 (13)	0.0011 (15)	−0.0072 (15)
C16	0.088 (3)	0.052 (2)	0.077 (2)	0.0129 (19)	0.009 (2)	−0.0090 (18)
C17	0.062 (2)	0.0402 (16)	0.071 (2)	0.0004 (14)	0.0133 (17)	−0.0029 (14)
C18	0.104 (3)	0.0449 (19)	0.083 (3)	0.005 (2)	0.009 (2)	0.0092 (18)
C19	0.0402 (15)	0.0411 (15)	0.0630 (17)	−0.0036 (12)	0.0048 (13)	0.0051 (13)
C20	0.0445 (16)	0.0382 (15)	0.0722 (19)	−0.0046 (12)	0.0036 (14)	−0.0062 (14)
C21	0.055 (2)	0.0498 (18)	0.078 (2)	0.0039 (16)	−0.0041 (17)	−0.0032 (16)
C22	0.066 (3)	0.056 (2)	0.126 (4)	0.0054 (19)	−0.030 (3)	−0.011 (2)
C23	0.043 (2)	0.069 (3)	0.148 (5)	0.0080 (17)	−0.008 (3)	−0.009 (3)
C24	0.052 (2)	0.083 (3)	0.135 (5)	0.004 (2)	0.031 (3)	−0.012 (3)
C25	0.056 (2)	0.060 (2)	0.091 (3)	−0.0015 (17)	0.0156 (19)	0.001 (2)
C26	0.0369 (15)	0.0466 (16)	0.0641 (17)	−0.0015 (12)	0.0024 (13)	0.0020 (14)
C27	0.0502 (19)	0.0510 (19)	0.083 (2)	0.0040 (15)	0.0034 (17)	0.0045 (17)
C28	0.058 (2)	0.0470 (19)	0.113 (3)	0.0028 (16)	0.003 (2)	0.007 (2)
C29	0.070 (3)	0.049 (2)	0.126 (4)	−0.0124 (18)	0.006 (3)	−0.014 (2)
C30	0.089 (3)	0.093 (3)	0.088 (3)	−0.035 (3)	0.002 (3)	−0.015 (3)
C31	0.063 (2)	0.070 (2)	0.070 (2)	−0.0136 (18)	−0.0083 (18)	−0.0008 (18)
C32	0.0362 (15)	0.0483 (17)	0.0624 (17)	0.0014 (12)	0.0060 (13)	0.0011 (14)
C33	0.0456 (17)	0.0442 (16)	0.073 (2)	0.0081 (13)	0.0025 (15)	−0.0023 (14)
C34	0.0499 (18)	0.0363 (15)	0.085 (2)	−0.0017 (13)	0.0023 (16)	0.0065 (15)
C35	0.062 (2)	0.079 (3)	0.071 (2)	−0.014 (2)	−0.0045 (18)	−0.005 (2)
C36	0.108 (4)	0.129 (5)	0.069 (2)	−0.034 (3)	0.008 (3)	−0.017 (3)
N1	0.0457 (14)	0.0392 (13)	0.0574 (14)	0.0019 (10)	−0.0038 (11)	−0.0014 (11)
N2	0.0589 (18)	0.0402 (15)	0.081 (2)	0.0012 (12)	−0.0044 (16)	−0.0011 (13)
N3	0.0396 (13)	0.0426 (13)	0.0622 (14)	0.0000 (10)	0.0053 (11)	0.0027 (11)
N4	0.0577 (18)	0.0550 (18)	0.0772 (19)	0.0000 (15)	0.0121 (15)	−0.0127 (15)
O1	0.0695 (16)	0.0490 (13)	0.0834 (16)	−0.0016 (12)	−0.0239 (14)	0.0030 (12)
O2	0.0626 (17)	0.0519 (15)	0.106 (2)	−0.0026 (12)	0.0136 (15)	−0.0058 (14)
O3	0.0511 (13)	0.0584 (14)	0.0791 (16)	−0.0047 (11)	0.0173 (12)	−0.0021 (12)
O4	0.0760 (19)	0.094 (2)	0.0821 (18)	0.0198 (17)	−0.0026 (16)	0.0077 (17)
S1	0.0985 (8)	0.0656 (6)	0.0537 (4)	0.0074 (5)	0.0004 (5)	−0.0062 (4)
S2	0.0592 (5)	0.0476 (5)	0.0805 (6)	−0.0027 (4)	0.0059 (4)	0.0147 (4)
O5C	0.059 (7)	0.114 (11)	0.103 (9)	0.018 (7)	0.012 (6)	−0.027 (8)
O5B	0.172 (12)	0.172 (12)	0.47 (4)	0.015 (16)	−0.096 (18)	−0.096 (18)

### Geometric parameters (Å, º)

**Table:**

O5A—O5A^i^	1.33 (4)	C19—C20	1.515 (4)
C1—C2	1.363 (5)	C19—S2	1.822 (3)
C1—C6	1.391 (5)	C19—H19	0.9800
C1—N1	1.439 (4)	C20—C21	1.379 (5)
C2—C3	1.415 (6)	C20—C25	1.384 (5)
C2—H2	0.9300	C21—C22	1.377 (6)
C3—C4	1.363 (8)	C21—H21	0.9300
C3—H3	0.9300	C22—C23	1.384 (8)
C4—C5	1.352 (8)	C22—H22	0.9300
C4—H4	0.9300	C23—C24	1.333 (7)
C5—C6	1.385 (6)	C23—H23	0.9300
C5—H5	0.9300	C24—C25	1.384 (7)
C6—H6	0.9300	C24—H24	0.9300
C7—N1	1.477 (4)	C25—H25	0.9300
C7—C8	1.496 (5)	C26—C31	1.385 (5)
C7—S1	1.827 (4)	C26—C27	1.388 (5)
C7—H7	0.9800	C26—N3	1.449 (4)
C8—C13	1.377 (6)	C27—C28	1.379 (6)
C8—C9	1.388 (5)	C27—H27	0.9300
C9—C10	1.440 (7)	C28—C29	1.378 (7)
C9—H9	0.9300	C28—H28	0.9300
C10—C11	1.321 (8)	C29—C30	1.376 (7)
C10—H10	0.9300	C29—H29	0.9300
C11—C12	1.322 (8)	C30—C31	1.392 (6)
C11—H11	0.9300	C30—H30	0.9300
C12—C13	1.386 (7)	C31—H31	0.9300
C12—H12	0.9300	C32—O3	1.208 (4)
C13—H13	0.9300	C32—N3	1.382 (4)
C14—O1	1.225 (4)	C32—C33	1.535 (5)
C14—N1	1.363 (4)	C33—N4	1.446 (5)
C14—C15	1.505 (5)	C33—C34	1.516 (5)
C15—N2	1.444 (5)	C33—H33	0.9800
C15—C16	1.525 (5)	C34—S2	1.796 (4)
C15—H15	0.9800	C34—H34A	0.9700
C16—S1	1.814 (4)	C34—H34B	0.9700
C16—H16A	0.9700	C35—O4	1.232 (5)
C16—H16B	0.9700	C35—N4	1.340 (6)
C17—O2	1.236 (5)	C35—C36	1.510 (6)
C17—N2	1.346 (5)	C36—H36A	0.9600
C17—C18	1.486 (6)	C36—H36B	0.9600
C18—H18A	0.9600	C36—H36C	0.9600
C18—H18B	0.9600	N2—H2N	0.87 (6)
C18—H18C	0.9600	N4—H4N	0.90 (4)
C19—N3	1.464 (4)		
			
C2—C1—C6	120.0 (3)	C21—C20—C19	122.6 (3)
C2—C1—N1	120.1 (3)	C25—C20—C19	118.8 (3)
C6—C1—N1	119.9 (3)	C22—C21—C20	119.9 (4)
C1—C2—C3	118.6 (4)	C22—C21—H21	120.1
C1—C2—H2	120.7	C20—C21—H21	120.1
C3—C2—H2	120.7	C21—C22—C23	120.5 (5)
C4—C3—C2	121.2 (5)	C21—C22—H22	119.7
C4—C3—H3	119.4	C23—C22—H22	119.7
C2—C3—H3	119.4	C24—C23—C22	119.7 (4)
C5—C4—C3	119.3 (4)	C24—C23—H23	120.2
C5—C4—H4	120.4	C22—C23—H23	120.2
C3—C4—H4	120.4	C23—C24—C25	120.8 (4)
C4—C5—C6	121.2 (4)	C23—C24—H24	119.6
C4—C5—H5	119.4	C25—C24—H24	119.6
C6—C5—H5	119.4	C24—C25—C20	120.4 (5)
C5—C6—C1	119.7 (4)	C24—C25—H25	119.8
C5—C6—H6	120.1	C20—C25—H25	119.8
C1—C6—H6	120.1	C31—C26—C27	120.3 (3)
N1—C7—C8	115.7 (3)	C31—C26—N3	119.3 (3)
N1—C7—S1	109.0 (2)	C27—C26—N3	120.4 (3)
C8—C7—S1	109.9 (2)	C28—C27—C26	119.8 (4)
N1—C7—H7	107.3	C28—C27—H27	120.1
C8—C7—H7	107.3	C26—C27—H27	120.1
S1—C7—H7	107.3	C29—C28—C27	120.2 (4)
C13—C8—C9	119.3 (4)	C29—C28—H28	119.9
C13—C8—C7	118.9 (4)	C27—C28—H28	119.9
C9—C8—C7	121.8 (3)	C30—C29—C28	120.3 (4)
C8—C9—C10	116.8 (4)	C30—C29—H29	119.9
C8—C9—H9	121.6	C28—C29—H29	119.9
C10—C9—H9	121.6	C29—C30—C31	120.2 (4)
C11—C10—C9	121.2 (5)	C29—C30—H30	119.9
C11—C10—H10	119.4	C31—C30—H30	119.9
C9—C10—H10	119.4	C26—C31—C30	119.3 (4)
C10—C11—C12	121.8 (5)	C26—C31—H31	120.4
C10—C11—H11	119.1	C30—C31—H31	120.4
C12—C11—H11	119.1	O3—C32—N3	120.0 (3)
C11—C12—C13	120.0 (5)	O3—C32—C33	120.1 (3)
C11—C12—H12	120.0	N3—C32—C33	119.7 (3)
C13—C12—H12	120.0	N4—C33—C34	112.9 (3)
C8—C13—C12	120.8 (5)	N4—C33—C32	109.4 (3)
C8—C13—H13	119.6	C34—C33—C32	116.3 (3)
C12—C13—H13	119.6	N4—C33—H33	105.8
O1—C14—N1	123.4 (3)	C34—C33—H33	105.8
O1—C14—C15	122.1 (3)	C32—C33—H33	105.8
N1—C14—C15	114.5 (3)	C33—C34—S2	108.6 (3)
N2—C15—C14	110.3 (3)	C33—C34—H34A	110.0
N2—C15—C16	111.1 (3)	S2—C34—H34A	110.0
C14—C15—C16	109.9 (3)	C33—C34—H34B	110.0
N2—C15—H15	108.5	S2—C34—H34B	110.0
C14—C15—H15	108.5	H34A—C34—H34B	108.3
C16—C15—H15	108.5	O4—C35—N4	121.3 (4)
C15—C16—S1	112.7 (2)	O4—C35—C36	121.8 (5)
C15—C16—H16A	109.1	N4—C35—C36	116.8 (5)
S1—C16—H16A	109.1	C14—N1—C1	120.3 (3)
C15—C16—H16B	109.1	C14—N1—C7	117.3 (3)
S1—C16—H16B	109.1	C1—N1—C7	122.4 (3)
H16A—C16—H16B	107.8	C17—N2—C15	122.7 (3)
O2—C17—N2	119.3 (3)	C17—N2—H2N	126 (4)
O2—C17—C18	123.2 (3)	C15—N2—H2N	111 (4)
N2—C17—C18	117.5 (4)	C32—N3—C26	118.4 (3)
N3—C19—C20	114.2 (3)	C32—N3—C19	128.0 (3)
N3—C19—S2	112.6 (2)	C26—N3—C19	113.5 (2)
C20—C19—S2	111.1 (2)	C35—N4—C33	122.7 (3)
N3—C19—H19	106.1	C35—N4—H4N	135 (2)
C20—C19—H19	106.1	C33—N4—H4N	102 (2)
S2—C19—H19	106.1	C16—S1—C7	97.23 (17)
C21—C20—C25	118.6 (3)	C34—S2—C19	95.94 (15)
			
C6—C1—C2—C3	−2.1 (5)	C29—C30—C31—C26	0.5 (7)
N1—C1—C2—C3	178.6 (3)	O3—C32—C33—N4	36.7 (4)
C1—C2—C3—C4	2.6 (7)	N3—C32—C33—N4	−147.8 (3)
C2—C3—C4—C5	−1.7 (7)	O3—C32—C33—C34	166.0 (3)
C3—C4—C5—C6	0.2 (7)	N3—C32—C33—C34	−18.5 (4)
C4—C5—C6—C1	0.2 (6)	N4—C33—C34—S2	−177.7 (2)
C2—C1—C6—C5	0.8 (5)	C32—C33—C34—S2	54.8 (3)
N1—C1—C6—C5	−179.9 (3)	O1—C14—N1—C1	−1.1 (5)
N1—C7—C8—C13	106.9 (4)	C15—C14—N1—C1	177.6 (3)
S1—C7—C8—C13	−129.2 (3)	O1—C14—N1—C7	179.9 (3)
N1—C7—C8—C9	−74.5 (4)	C15—C14—N1—C7	−1.3 (4)
S1—C7—C8—C9	49.4 (4)	C2—C1—N1—C14	−62.4 (4)
C13—C8—C9—C10	−2.3 (6)	C6—C1—N1—C14	118.4 (4)
C7—C8—C9—C10	179.1 (3)	C2—C1—N1—C7	116.5 (4)
C8—C9—C10—C11	0.7 (7)	C6—C1—N1—C7	−62.7 (4)
C9—C10—C11—C12	−0.2 (8)	C8—C7—N1—C14	−174.3 (3)
C10—C11—C12—C13	1.3 (9)	S1—C7—N1—C14	61.3 (3)
C9—C8—C13—C12	3.5 (6)	C8—C7—N1—C1	6.7 (4)
C7—C8—C13—C12	−177.9 (4)	S1—C7—N1—C1	−117.7 (3)
C11—C12—C13—C8	−3.0 (8)	O2—C17—N2—C15	−2.4 (6)
O1—C14—C15—N2	−6.0 (5)	C18—C17—N2—C15	177.4 (3)
N1—C14—C15—N2	175.3 (3)	C14—C15—N2—C17	−152.0 (3)
O1—C14—C15—C16	116.8 (4)	C16—C15—N2—C17	85.9 (4)
N1—C14—C15—C16	−61.9 (4)	O3—C32—N3—C26	−4.8 (5)
N2—C15—C16—S1	179.1 (3)	C33—C32—N3—C26	179.7 (3)
C14—C15—C16—S1	56.7 (4)	O3—C32—N3—C19	177.3 (3)
N3—C19—C20—C21	−34.8 (4)	C33—C32—N3—C19	1.8 (5)
S2—C19—C20—C21	93.9 (3)	C31—C26—N3—C32	117.9 (4)
N3—C19—C20—C25	147.0 (3)	C27—C26—N3—C32	−64.9 (4)
S2—C19—C20—C25	−84.3 (3)	C31—C26—N3—C19	−63.9 (4)
C25—C20—C21—C22	2.0 (5)	C27—C26—N3—C19	113.2 (3)
C19—C20—C21—C22	−176.3 (3)	C20—C19—N3—C32	104.5 (4)
C20—C21—C22—C23	0.0 (6)	S2—C19—N3—C32	−23.5 (4)
C21—C22—C23—C24	−2.2 (7)	C20—C19—N3—C26	−73.5 (3)
C22—C23—C24—C25	2.4 (8)	S2—C19—N3—C26	158.6 (2)
C23—C24—C25—C20	−0.4 (7)	O4—C35—N4—C33	1.1 (6)
C21—C20—C25—C24	−1.8 (6)	C36—C35—N4—C33	−177.4 (4)
C19—C20—C25—C24	176.5 (4)	C34—C33—N4—C35	−74.5 (4)
C31—C26—C27—C28	−1.9 (6)	C32—C33—N4—C35	56.6 (4)
N3—C26—C27—C28	−179.0 (3)	C15—C16—S1—C7	−1.7 (4)
C26—C27—C28—C29	1.3 (6)	N1—C7—S1—C16	−53.5 (3)
C27—C28—C29—C30	0.2 (7)	C8—C7—S1—C16	178.7 (3)
C28—C29—C30—C31	−1.1 (7)	C33—C34—S2—C19	−65.8 (2)
C27—C26—C31—C30	1.0 (6)	N3—C19—S2—C34	50.5 (3)
N3—C26—C31—C30	178.1 (4)	C20—C19—S2—C34	−79.1 (2)

Symmetry code: (i) −*y*, −*x*, −*z*+1/2.

### Hydrogen-bond geometry (Å, º)

**Table:**

*D*—H···*A*	*D*—H	H···*A*	*D*···*A*	*D*—H···*A*
N2—H2*N*···O1	0.88 (6)	2.22 (5)	2.678 (4)	113 (5)
N4—H4*N*···O2	0.90 (4)	2.03 (4)	2.899 (5)	162 (4)
C7—H7···O3	0.98	2.50	3.241 (4)	132
C21—H21···O4	0.93	2.48	3.375 (5)	162
